# A lake-centric geospatial database to guide research and inform management decisions in an Arctic watershed in northern Alaska experiencing climate and land-use changes

**DOI:** 10.1007/s13280-017-0915-9

**Published:** 2017-03-25

**Authors:** Benjamin M. Jones, Christopher D. Arp, Matthew S. Whitman, Debora Nigro, Ingmar Nitze, John Beaver, Anne Gädeke, Callie Zuck, Anna Liljedahl, Ronald Daanen, Eric Torvinen, Stacey Fritz, Guido Grosse

**Affiliations:** 1 U.S. Geological Survey, Alaska Science Center, 4210 University Drive, Anchorage, AK 99508 USA; 2 Water and Environmental Research Center, University of Alaska Fairbanks, 467 Duckering Avenue, Fairbanks, AK 99775 USA; 3 Bureau of Land Management, Arctic Field Office, 222 University Avenue, Fairbanks, AK 99709 USA; 4 Alfred Wegener Institute Helmholtz Centre for Polar and Marine Research, Telegrafenberg A43, 14469 Potsdam, Germany; 5 Department of Geography, University of Potsdam, Potsdam, Germany; 6 BSA Environmental Services, Inc., 23400 Mercantile Rd. #8, Beachwood, OH 44122 USA; 7 Alaska Department of Natural Resources, Division of Geological & Geophysical Surveys, 3354 College Rd., Fairbanks, AK 9907 USA; 8 School of Fisheries and Ocean Sciences, University of Alaska Fairbanks, 902 Koyukuk Ave., Fairbanks, AK 99775 USA; 9 Institute of Earth and Environmental Science, University of Potsdam, Telegrafenberg A43, 14473 Potsdam, Germany

**Keywords:** Arctic, Climate Change, GIS, Lakes, Land-use change, Watershed

## Abstract

Lakes are dominant and diverse landscape features in the Arctic, but conventional land cover classification schemes typically map them as a single uniform class. Here, we present a detailed lake-centric geospatial database for an Arctic watershed in northern Alaska. We developed a GIS dataset consisting of 4362 lakes that provides information on lake morphometry, hydrologic connectivity, surface area dynamics, surrounding terrestrial ecotypes, and other important conditions describing Arctic lakes. Analyzing the geospatial database relative to fish and bird survey data shows relations to lake depth and hydrologic connectivity, which are being used to guide research and aid in the management of aquatic resources in the National Petroleum Reserve in Alaska. Further development of similar geospatial databases is needed to better understand and plan for the impacts of ongoing climate and land-use changes occurring across lake-rich landscapes in the Arctic.

**Electronic supplementary material:**

The online version of this article (doi:10.1007/s13280-017-0915-9) contains supplementary material, which is available to authorized users.

## Introduction

Roughly 25% of the global lake population occurs in the northern high-latitude region (Lehner and Döll 2004). The skewed global distribution of lakes toward the Arctic can be explained primarily by glaciation history and the presence of permafrost (Smith et al. 2007). In Arctic lowland regions with ice-rich permafrost, thermokarst lakes may account for as much as 20–40% of the land surface (Grosse et al. 2013) and drained lake basins (the sites of former lakes) may account for an additional 40–75% of the landscape (Hinkel et al. 2005; Grosse et al. 2006; Jones et al. 2012; Jones and Arp 2015). These abundant landscape features provide habitat for a variety of migratory waterfowl during the open-water season (Haynes et al. 2014a) and fish species year-round (Morris 2003; Morris et al. 2006; Reist et al. 2006; Haynes et al. 2014b; Laske et al. 2016), are an important component of the northern latitude carbon cycle (Wik et al. 2016), and factor prominently into regional hydrology (Bowling et al. 2003; Arp et al. 2012a). Lakes also provide a source of water for remote Arctic communities (Martin et al. 2007; Alessa et al. 2008) as well as oil and gas exploration activities (Sibley et al. 2008; Jones et al. 2009). Due to the importance of lakes in the Arctic, better representation of their characteristics and variability across the landscape is necessary because they are vulnerable to both natural and anthropogenic stressors (Vorosmarty et al. 2000; Hinkel et al. 2007; Williamson et al. 2009).

Land cover classification schemes are useful for categorically describing often complex and heterogeneous natural and human-modified landscapes (Anderson 1976). In northern Alaska, several raster- and vector-based land cover classifications have been developed in recent decades that largely focus on describing tundra vegetation communities (Markon and Derksen 1994; Muller et al. 1998; BLM-Alaska 2002; Jorgenson and Heiner 2003; Walker et al. 2005; NSSI 2013). In particular, two relatively recent land cover classifications provide detailed information as mapped at a 30 m spatial resolution across the ~250 000 km^2^ area encompassing the North Slope of Alaska (Jorgenson and Heiner 2003; NSSI 2013). However, these classification schemes provide scant information on surface water features, typically designating them as open water or at best classifying them as either lacustrine or riverine. Owing to the diversity of surface water features on the landscape, particularly in lakes and lake types that vary hydrologically, morphometrically, and chemically, more detailed assessments of these pervasive land cover feature types are needed.

Due to the abundance of lakes in Arctic Alaska, several studies have focused on describing regional lake classes based on density and limnicity (lake surface area relative to land surface area) (Sellmann et al. 1975; Hinkel et al. 2005, Arp and Jones 2009), surface area dynamics (Hinkel et al. 2007; Jones et al. 2009), lake depth (Mellor 1987; Kozlenko and Jeffries 2000; Hinkel et al. 2012a; Grunblatt and Atwood 2014), salinity (Arp et al. 2010), water temperature regimes (Hinkel et al. 2012b), and lake ice regimes (Arp et al. 2012b; Jones et al. 2013; Surdu et al. 2014). While each of these classification systems provides useful information to assess research and management needs regarding specific aspects such as water supply, habitat provision, or vulnerability to drought or drainage, none provide comprehensive information encompassing integrated management needs surrounding Arctic lakes. Additionally, although previous Alaska lake classifications and databases have utilized natural domains such as lake districts (Arp and Jones 2009) or physiographic provinces (Sellmann et al. 1975; Hinkel et al. 2005), none focus on natural hydrologic units (i.e., watersheds), which have direct relevance to hydrology, water supply, and fisheries management.

**Fig. 1:**
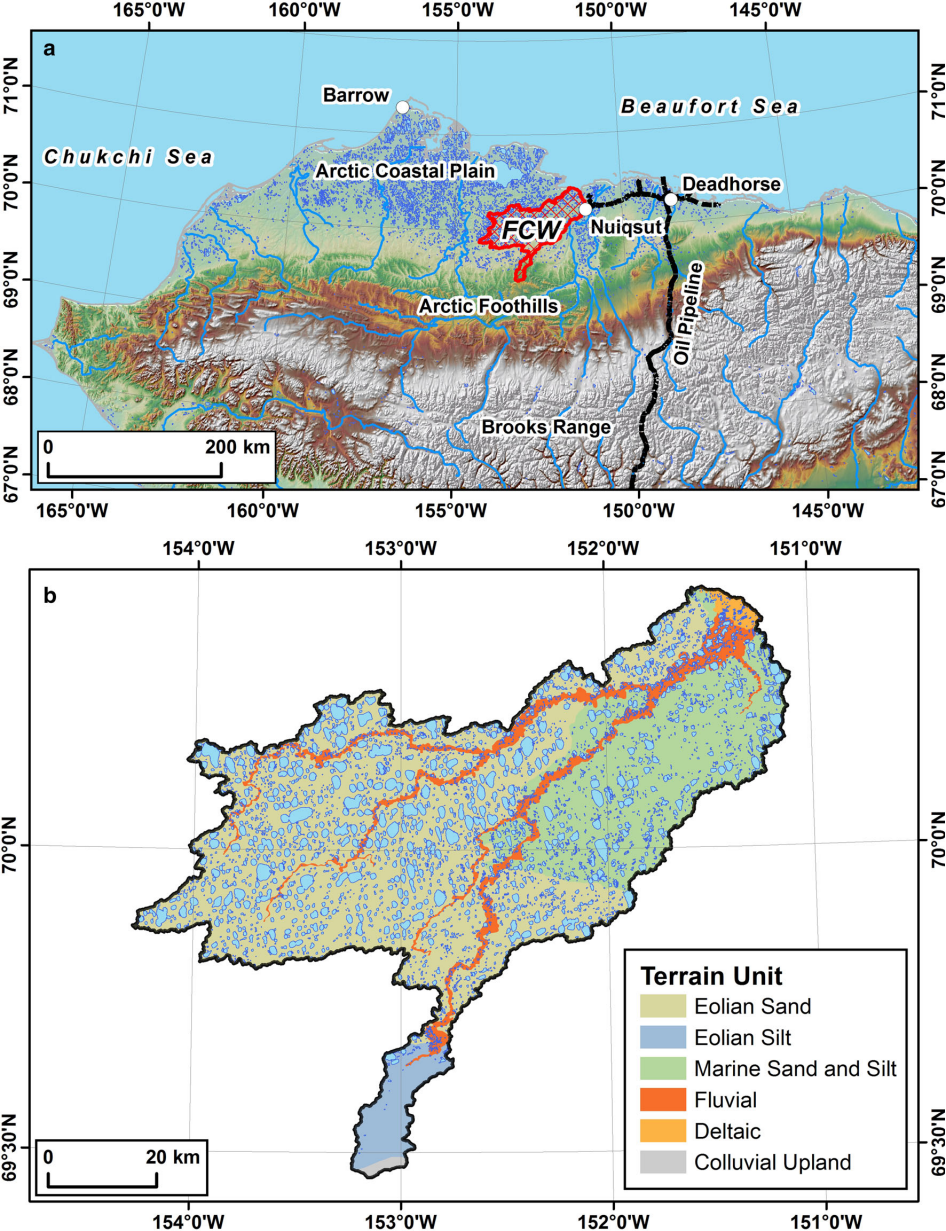
Study area figure. **a** Hillshade relief map of northern Alaska showing the location of the Fish Creek Watershed (*red hatched polygon*). **b** Terrain units of the Fish Creek Watershed along with lakes from the lake-centric geospatial database shown as *blue polygons*

A recent analysis of the 4900 km^2^ Fish Creek Watershed (FCW), located entirely within the National Petroleum Reserve in Alaska (NPR-A), focused on understanding landscape heterogeneity in freshwater ecosystems and the linkage to physical drivers (Arp et al. 2012b) by capitalizing on existing geospatial and monitoring datasets as part of the Fish Creek Watershed Observatory (FCWO) (Whitman et al. 2011). The sub-watersheds of the FCW represent a natural physiographic gradient of deltaic, lacustrine, riverine, and eolian landforms which, combined with more than a decade of climate and river discharge observations, make this landscape an ideal setting to advance our understanding of Arctic landscape processes. The FCW is particularly important for the subsistence harvest of fish, caribou, and geese for the predominantly Iñupiat community of Nuiqsut which is located just outside of the FCW. Commercial petroleum development in the NPR-A began in the lower FCW in 2015 and is expected to progress further into the watershed during the next decade (USDOI BLM 2015). For the first time since the establishment of the NPR-A in the 1970s, the construction of permanent infrastructure including water supply for winter ice roads and pads to support development is necessary. In response to this planned development, the Bureau of Land Management (BLM), U.S. Geological Survey (USGS), and University of Alaska Fairbanks (UAF) have been collecting baseline datasets to describe natural variability of freshwater ecosystems in order to assess any impacts of development activities, such as lake water extraction, temporary and permanent roads, drilling pads, or increases in dust or contaminants. Science applied toward industrial (oil and gas) development and subsistence harvest concerns in the lower FCW has similarly advanced our understanding of fish distribution and migration (Morris 2003; Heim et al. 2015), food web dynamics (McFarland 2015), and lake (Jorgenson and Shur 2007), stream (Arp et al. 2015a), and permafrost processes (Jorgenson et al. 2006). In this study, we develop a lake-centric geospatial database for the FCW (Fig. 1), an Arctic watershed in northern Alaska that is currently experiencing climate and land-use change pressures, which will guide research and aid in the management of the natural and human-modified Arctic environment. The geospatial dataset along with detailed metadata are available at http://alaska.usgs.gov/products/data.php?dataid=78 (Jones and Zuck 2016).

## Materials and methods

High-resolution aerial photography and airborne Interferometric Synthetic Aperture Radar (IfSAR) data were acquired throughout the NPR-A between 2002 and 2006. The area covering the FCW was acquired in 2002. Digital surface and terrain models were derived from the IfSAR data and used to create an orthorectified aerial photography dataset at the spatial resolution of 5 and 2.5 m, respectively. These high-quality geospatial datasets provided the framework for delineation of the FCW and development of a contemporary lakes data layer that was used as the basis for our lake-centric geospatial database (Fig. S1). Geospatial information pertaining to lake geometry, lake type, surface elevation, bluff height characteristics, surficial geology, hydrologic connectivity, depth, landscape position, dominant vegetation classes present in the vicinity of a lake, and other attributes assigned to the dataset are described in detail below.

### Watershed delineation and lake extraction

The FCW was derived from the IfSAR digital surface model (DSM) data using the hydrology toolset in ArcGIS 10.1. This process followed the inherent workflow available in the toolbox. This involved filling sinks in the DSM data, computation of a flow direction raster, calculation of a flow accumulation raster, and placement of pour points to use as the mouth of the primary and sub-watersheds. The watershed delineation was then built on the flow direction, flow accumulation, and pour points.

Surface water features were extracted from the IfSAR DSM by extracting all zero-slope landscape features in the watershed. As a result of “hydro-flattening” by the vendor during raw data processing, calculation of a slope derivative layer provides an initial approximation of waterbodies for the study area. However, this technique for extracting waterbodies from the DSM inherently underestimates the entire perimeter of a lake by 1 pixel since the edge pixel always represents a non-zero slope. To account for this underestimation in lake area, the expand feature in ArcGIS^®^ was used to automatically extend the perimeter of every waterbody by 1 pixel. This binary raster file (water and non-water pixels) was then exported as a polygon vector for cleanup and analysis. All non-lake polygons were manually removed from the dataset by overlaying the polygon layer on the CIR orthophoto mosaic and using a scale of 1:24 000. Once lakes had been isolated in the polygon data layer, this was converted back to a 5-m resolution raster file for further editing. During this phase, the performance of the IfSAR-extracted lake layer was assessed using the CIR orthophoto mosaic resampled to a 5-m spatial resolution. In instances where there was a discrepancy in the lake surface area between the two datasets, the perimeter of the lake polygon was manually adjusted to match that of the CIR photos in raster space using the ArcScan tool in ArcMap^®^. This technique retained the raster grid network resolution (5 m) for depicting surface water area. After this step was complete, the binary raster lake layer was converted back to a polygon vector layer and compared to the initial extraction of lakes in the FCW. This resulted in the manual correction of 408 of the 4362 lakes or 9% of the lakes in the watershed. This exercise also provided an indirect accuracy assessment by indicating that 91% of the lakes were accurately retrieved from the IfSAR DSM using the automated approach.

### Lake-centric geospatial database development

We developed a lake-centric geospatial database in a GIS environment based on information derived from the IfSAR DSM and CIR orthophotography, the availability of existing geospatial data, and observational field data (Table 1). This geospatial database consists of 22 descriptive attributes assigned to each lake vector polygon. Selection of these particular attributes and how these should be classified was determined iteratively among an interdisciplinary team consisting of both scientists and land managers with Arctic-specific expertise in fish and wildlife biology, hydrography and hydrology, permafrost and geomorphology, and Arctic climatology. A brief description of each attribute and attribute accuracies is given below.

**Table 1:** Data descriptions for the lake-centric geospatial database developed for the FCW. Attribute, data source, data resolution, data year, classification technique, and attribute accuracy

Attribute	Data source	Data resolution	Data year	Classification technique	Attribute accuracy
Latitude	IfSAR DSM	5 m	2002	Semi-automated	±2.5 m
Longitude	IfSAR DSM	5 m	2002	Semi-automated	±2.5 m
Perim_km	IfSAR DSM	5 m	2002	Semi-automated	±2.5 m
Area_sq_km	IfSAR DSM	5 m	2002	Semi-automated	91%
Hectares	IfSAR DSM	5 m	2002	Semi-automated	91%
Shape_ind	IfSAR DSM	5 m	2002	Semi-automated	N/A
Elev_masl	IfSAR DSM	5 m	2002	Semi-automated	±1.0 m
Surf_geo	Jorgenson and Grunblatt (2013)	1:300 000	2013	Manual Interpretation	N/A
Lake_type	CIR Orthophotography	2.5 m	2002	Manual interpretation	N/A
Depth_cat	Grunblatt and Atwood (2014)	12.5 m	2009	Automated	89%
Unfrz_perc	Grunblatt and Atwood (2014)	12.5 m	2009	Automated	74–98%
Rel_depth	CIR Orthophotography	2.5 m	2002	Manual interpretation	N/A
Connectiv	IfSAR DSM	5 m	2002	Automated	73%
Mean_b_hgt	IfSAR DSM	5 m	2002	Automated	±1.0 m
Min_b_hgt	IfSAR DSM	5 m	2002	Automated	±1.0 m
Max_b_hgt	IfSAR DSM	5 m	2002	Automated	±1.0 m
100m_gradi	IfSAR DSM	5 m	2002	Automated	±1.0 m
Dist_cst	IfSAR DSM	5 m	2002	Automated	±5.0 m
Islands	CIR Orthophotography	2.5 m	2002	Manual interpretation	N/A
Emerg_veg	CIR Orthophotography	2.5 m	2002	Manual interpretation	N/A
Dom_ecosys	Jorgenson and Heiner (2003)	30 m	ca. 2000	Automated	~60–70%
LSAD	Landsat TM, ETM + OLI	30 m	1985–2014	Automated	95%

#### Lake metrics

The IfSAR DSM was the basis for the lake vector polygon dataset developed for the FCW which provided additional useful spatially consistent information. In addition to general information on the lake vector polygons such as the lake centroid coordinates (Longitude and Latitude), perimeter (Perim_km), surface area (Area_sq_km), and shape complexity (Shape_ind), the IfSAR data also provided information on lake surface elevation (Elev_m), the mean (Mean_b_hgt), minimum (Min_b_hgt), and maximum (Max_b_hgt) bluff heights surrounding each lake (5 m buffer), and the difference in elevation between the lake surface and the minimum elevation in a 100-m buffer around each lake (100 m_gradi). The spatial accuracy of the lake vector polygon layer is ±2.5 m, whereas the accuracy of the derived surface elevation information is ±1.0 m.

#### Relative lake depth

Relative lake depth information was assigned to each lake using the SAR-based classification scheme of bedfast and floating ice lakes developed by Grunblatt and Atwood (2014). The distinction between grounded and floating ice lakes provides information on whether a lake is relatively shallow or relatively deep (Depth_cat) and during 2009 (year of image acquisition in Grunblatt and Atwood classification) this cutoff occurred around 1.6 m for the FCW. The surficial percentage of floating ice (not grounded to bed) (Unfrz_perc) was also quantified for each lake based on the same data. The overall accuracy of this SAR-based classification scheme was 89% with the accuracy of point-specific retrievals of floating and grounded ice conditions ranged from 74 to 98% (Grunblatt and Atwood 2014). Since it is not possible to further distinguish deeper depths using this technique, we manually interpreted lakes with deep central pools using the CIR orthophotography. This interpretation was aided by a compilation of ~200 maximum lake depths from a number of sources in the watershed and roughly delineates lakes deeper than 4 m. In the end, we added a relative depth (rel_depth) attribute to the geospatial database indicating shallow (<1.6 m), intermediate (1.6–4.0 m), and deep lakes (>4.0 m). This information was based on expert judgement that was guided by field observations and data and thus we cannot provide a robust measure of the accuracy of this attribute.

#### Lake connectivity

The lake connectivity attribute (Connectiv) was computed based on the ArcGIS hydrology toolset and the IfSAR DSM data. Determination of lake connectivity classes involved a different set of rules for each respective surficial geology class or landscape setting (Table 2; Figure S1). The 0.5 km^2^ threshold and additional rule sets were determined primarily based on an intra-watershed synoptic survey of stream discharge during very low-flow conditions in mid-July 2015 to develop discharge–drainage area relationships for the major surface geology units (Table 2). Additional information on minimum flow conditions were derived from continuous discharge records (Whitman et al. 2011; Arp et al. 2015a) and fish migration studies (Heim et al. 2015) in the FCW. Comparison of this classification to field survey data collected at 22 lakes during the mid-summer of 2015 showed 73% accuracy. The majority of misclassifications occurred between the temporary and perennial connectivity categories due to limited measurements of low-flow conditions and for lakes set in alluvial marine silt surficial geology where low flow—drainage area relationship was more poorly defined (Figure S1).

**Table 2:** Summary of drainage area thresholds for major surficial geology units used to classify lake hydrologic connectivity to stream and river networks

Surficial geology	Isolated	Temporary connection	Perennial connection	Flow-through
Marine sand and silt	No intersection with a stream with a 0.5 km^2^ contributing area	Intersection with 15 km^2^ contributing area stream	Intersection with 25 km^2^ contributing area stream	Intersection with greater than 25 km^2^ contributing area stream
Eolian sand	No intersection with a stream with a 0.5 km^2^ contributing area	Intersection with 5 km^2^ contributing area stream	Intersection with 10 km^2^ contributing area stream	Intersection with greater than 10 km^2^ contributing area stream
Eolian silt	No intersection with a stream with a 0.5 km^2^ contributing area	Intersection with 50 km^2^ contributing area stream	Intersection with 100 km^2^ contributing area stream	Intersection with greater than 100 km^2^ contributing area stream
Floodplain	No intersection with a stream with a 0.5 km^2^ contributing area	Intersection with 0.5 km^2^ contributing area stream with a gradient greater than 0.5 m	Intersection with 0.5 km^2^ contributing area stream with a gradient less than 0.5 m	N/A
Delta	No intersection with a stream with a 0.5 km^2^ contributing area	Intersection with 0.5 km^2^ contributing area stream with a gradient greater than 0.5 m	Intersection with 0.5 km^2^ contributing area stream with a gradient less than 0.5 m	N/A

#### Lake peripheral land cover

Information on the dominant land cover type surrounding each lake was derived from the Northern Alaska Ecosystems map (Jorgenson and Heiner 2003). This land cover map consists of 36 distinct categories that provide information on broad vegetative classes, barren ground, surface water, and landscape setting. We extracted the dominant land cover type (DOM_Ecosys) within a 90-m buffer (3 land cover pixels) surrounding each lake in the database. This attribute in the geospatial database consists of 16 different peripheral land cover types (Jones and Zuck 2016; see SOM Data Dictionary). The overall classification accuracy of this product and the accuracy of individual classes are unknown. However, an approximate accuracy that ranges from 60 to 70% was provided and is based on similar Landsat-based land cover classification efforts in Alaska (personal communication M. Torre Jorgenson).

#### Lake surface area dynamics

Lake surface area dynamics were classified from a dense time series of Landsat TM, ETM+ , and OLI imagery available from 1985 to 2014. Trends of different multispectral indices were calculated for each pixel on all unobstructed observations during peak summer season (July, August) between 1985 and 2014 (Nitze and Grosse 2016). The number of observations per individual lake ranged from 45 to 115 and encompassed analysis of more than 500 Landsat scenes covering the FCW. The trend trajectories and magnitude, which indicate changes in vegetation, wetness, and other surface properties, were classified and spatially assigned to each lake polygon. Changes in lake surface area were categorized over the entire time series as (1) expanding lakes, (2) shrinking lakes, (3) dynamic lakes (expanding and shrinking), and (4) stable lakes. The ability to accurately distinguish between land and water pure pixels is 95%; however, changes occurring at the sub-pixel level were not evaluated.

#### Additional attributes

**Fig. 2:**
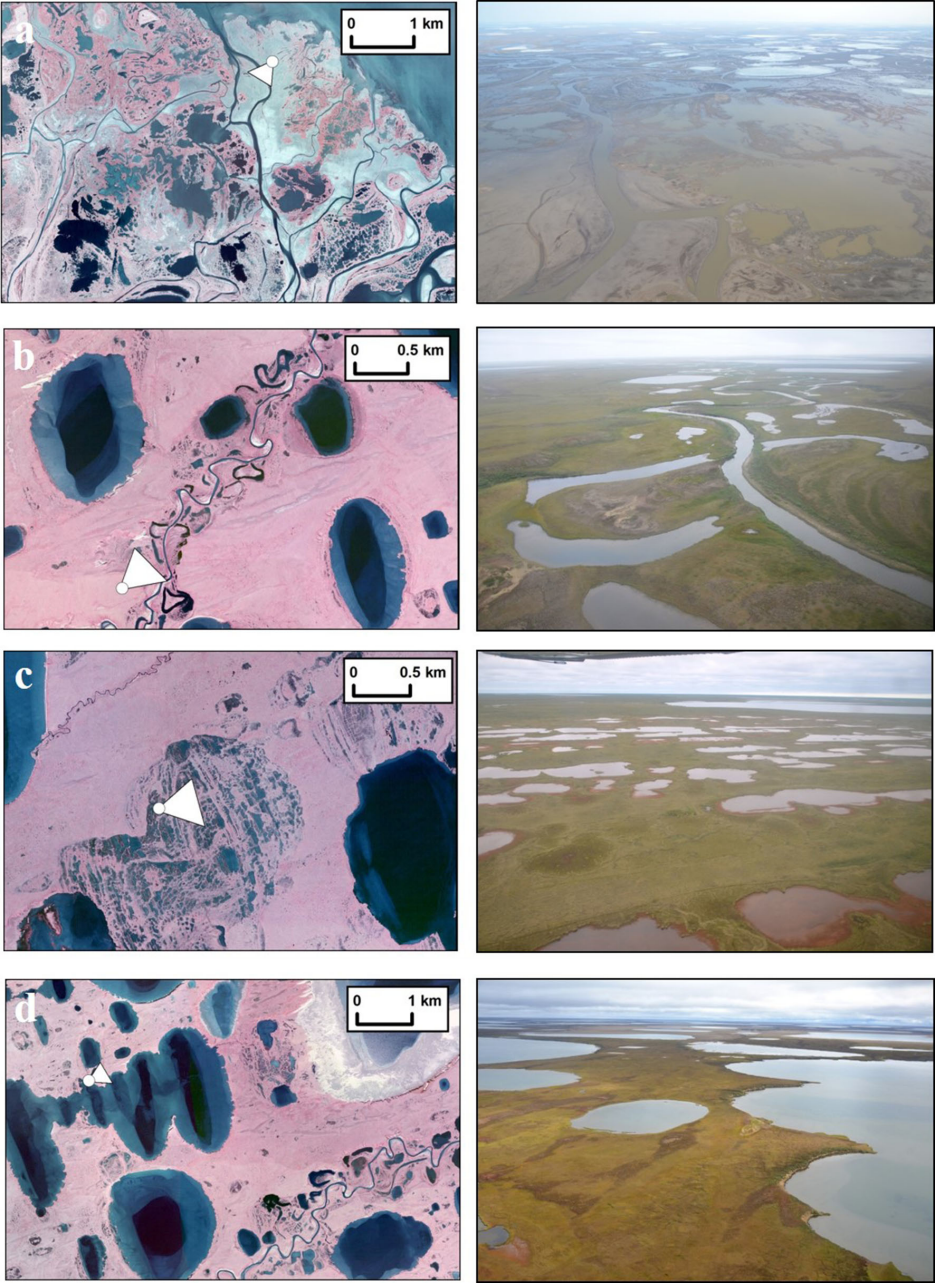
Vertical and oblique photos of various lake types in the watershed. **a** Deltaic lakes, **b** fluvial and depression lakes, **c** marginal ponds in drained lake basins, and **d** depression lakes located in the eolian sand region. The *white dot* represents the location of the oblique photo (*right*) and the *triangle* represents the perspective. Note differences in scale between **a** and **d** (*left*)

Lake types were manually interpreted and their classification aimed at describing lake origin and history (Fig. 2). Lakes were assigned to one of six types (lake type) using the CIR orthophotography and consisted of (1) marginal drained lake basin ponds, (2) primary or secondary thermokarst or depression lakes, (3) oxbow lakes, (4) deltaic lakes, (5) anthropogenic ponds, and (6) collapsed pingo ponds. The presence of islands (islands) in a lake and the presence of emergent vegetation (Emerg_veg; most likely *Arctophila fulva*) were also manually interpreted using the CIR orthophotography and included in the lake-centric geospatial database. We are not able to provide an accuracy statement for the manually interpreted attributes (lake type, lakes with islands, and lakes with emergent vegetation); however, each value was interpreted by the same person and at the same spatial scale to be as consistent as possible. Distance of a lake to the coast (Dist_cst) was also added to the attribute table and measured as a straight-line distance relative to the Fish Creek delta mouth and is accurate within ±5 m. Information regarding the underlying surficial geology comes from Jorgenson and Grunblatt (2013) and was mapped at a scale of 1:300 000.

#### Biological data collection

As part of ongoing research in the FCW, we have begun to collect biological data to characterize Arctic lake food webs that utilized the lake-centric geospatial database to achieve a balanced representation of lake habitats. In this study, we specifically present data from surveys of lake fish communities and loon species because they are of ongoing management interest. Fish community composition is of specific interest for managing winter water extraction from lakes with current regulations for extraction amounts based on which species are present. Data presented in this study were collected from 23 lakes during the summers of 2014 and 2015 using a multiple gear-type approach as outlined in Haynes et al. (2013). Loon data presented in this study are from aerial surveys conducted in late June 2015 following methods in Schmidt et al. (2014). For the purposes of this study, only presence data relative to lake classes are used to illustrate the potential of the watershed-based, lake-centric geospatial database to help understand biological issues of management concern.

## Results

The 4900 km^2^ FCW is composed of three primary watersheds: Fish Creek (2463 km^2^), Judy Creek (1725 km^2^), and the Ublutuoch River (663 km^2^). For our assessment, we also included the 49 km^2^ Pik Dunes watershed since it was initially part of the FCW before it partially drained catastrophically at some point in the past through natural processes, isolating it from the modern-day FCW system. Below, we describe in detail the characteristics of lakes based on their size, shape, landscape position, connectivity, type, depth, and dynamics.

### Lake distribution and surface area

We identified 4362 lakes with a surface area greater than 1 ha covering 19% of the FCW based on the IfSAR data acquired in mid-July 2002. The average lake size in the watershed is 20 ha and the largest lake in the watershed is 1070 ha. Lakes range in elevation from sea level to 140 m asl and are distributed across five primary terrain units: (1) eolian sand, (2) eolian silt, (3) marine sand and silt, (4) fluvial, and (5) deltaic (Table 3; Fig. 1b). Lake density is highest in the fluvial and deltaic settings (>2 lakes/km^2^), with less than one lake per square kilometer in the eolian sand (0.73 lakes/km^2^), eolian silt (0.21 lakes/km^2^), and marine sand and silt (0.91 lakes/km^2^) terrain units. Limnicity, however, is highest in the eolian sand unit (22%), but lowest in the eolian silt unit (3%). The limnicity of the marine sand and silt unit is 14%, the fluvial unit is 17%, and the deltaic unit is 10%.

**Table 3:** Summary of major lake geographic and geomorphic characteristics among lake type classes by number (No.) and area (A) of lakes as a percentage

Attribute	Remnant Pond		Thermokarst/Depression lake		Oxbow lake		Deltaic lake		All classes	
	No. (%)	A (%)	No. (%)	A (%)	No. (%)	A (%)	No. (%)	A (%)	No. (%)	A (%)
Lake type	35.9	4.6	45.3	90.3	16.8	4.7	2	0.4	100	100
Surface geology										
Delta	0	0	0.1	0	0	0	97.7	99.2	1.9	0.4
Floodplain	2.9	2.9	9.6	4	91.4	94.5	1.2	0.3	20.8	8.2
Eolian sand	42.4	37.3	74.4	77.2	6	3.5	0	0	50	71.6
Eolian silt	1.5	1.8	0.9	0.6	0	0	0	0	0.9	0.6
Marine sand	53.1	58	15	18.1	2.6	2	1.2	0.5	26.3	19.1
Depth class										
Shallow	93.7	85	18.1	3.8	47.5	16.4	100	100	51.8	8.5
Intermediate	6.3	15	47.2	31.4	52	82.7	0	0	32.4	32.9
Deep	0	0	34.7	64.8	0.5	0.9	0	0	15.8	58.6
Connectivity										
Isolated	59.1	47.4	32	5.3	63.7	34.2	82.6	52	48.1	8.8
Temporary	36.4	46.9	47.2	49	21.8	48	0	0	38.1	48.7
Perennial	2.4	3.1	10.6	20.1	13.9	17.4	17.4	48	8.3	19.3
Flow-through	2.1	2.6	10.2	25.6	0.5	0.4	0	0	5.5	23.3
Islands										
Present	44.2	52.6	15.2	32.5	19.9	33.6	68.6	80.2	27.4	33.6
Absent	55.8	47.4	84.8	67.5	80.1	66.4	31.4	19.8	72.6	66.4
Emergent vegetation										
Present	3.5	7.3	12	26.5	5.5	8.5	0	0	7.6	24.6
Absent	96.5	92.7	88	73.5	94.5	91.5	100	100	92.4	75.4

### Lake type

**Fig. 3:**
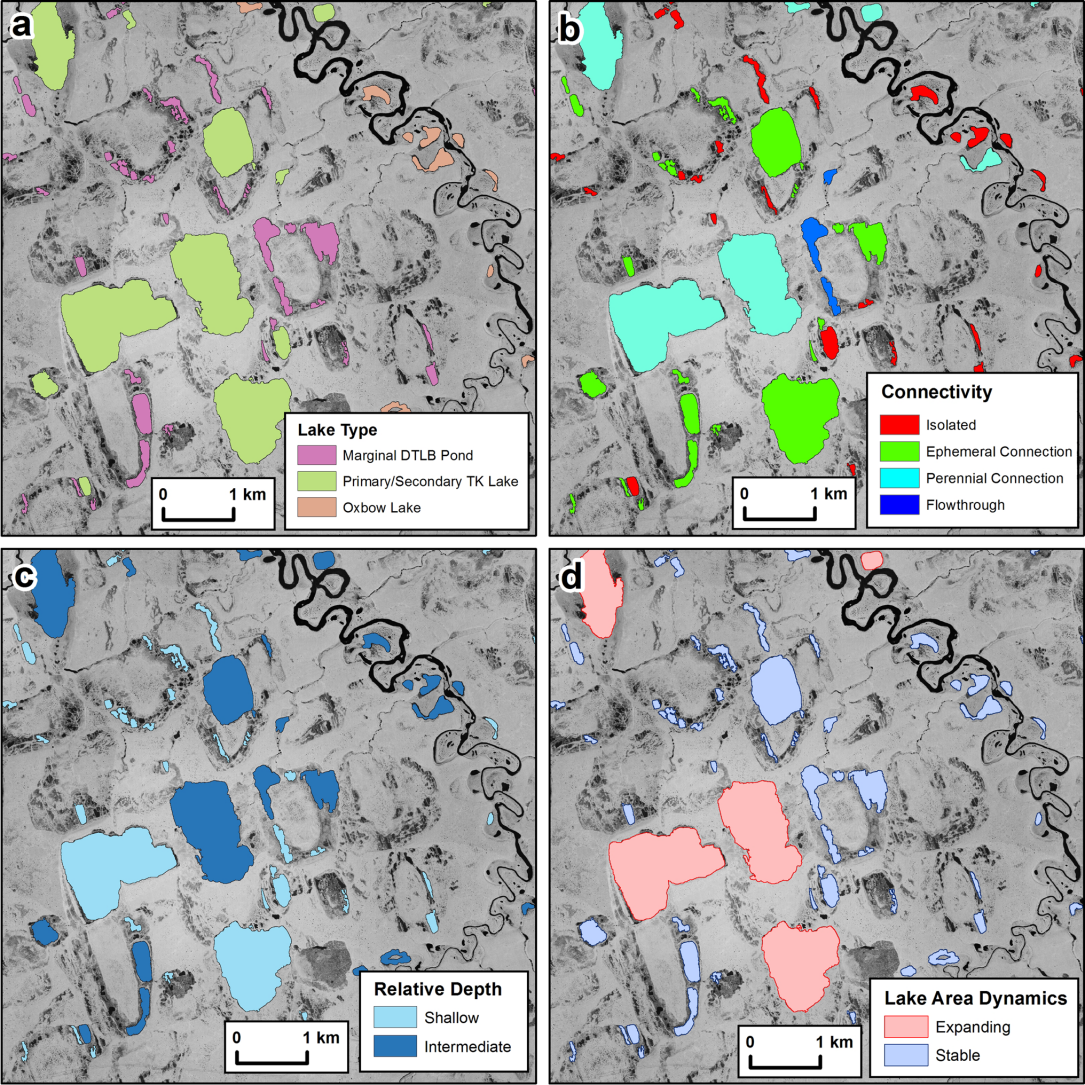
Select attributes from the lake-centric geospatial database for a location in the lower portion of the Fish Creek Watershed: **a** lake type, **b** hydrologic connectivity, **c** relative lake depth, and **d** lake area dynamics

We identified six general lake types in the FCW (Table 3; Figs. 2, 3a). Primary (first-generation) and/or secondary (later generation) thermokarst and depression (lying within low ground-ice content rolling sand dune topography) lakes accounted for 45.3% of lakes and 90.3% of lake surface area in the watershed (Table 3). Remnant ponds that flank the margins of drained lake basins accounted for 35.9% of lakes in the watershed but only 4.6% of total surface area. Oxbow lakes accounted for 16.8% of the number and 4.7% of the lake surface area, whereas deltaic lakes accounted for 2.0 and 0.4%, respectively. There was one human created pond and one collapsed pingo pond that exceeded 1 ha in the watershed and accounted for less than 0.1% of both the number and area of lakes in the watershed and not included in Table 3.

### Lake depth

Of the 4362 lakes larger than 1 ha in the FCW, 52% are classified as having bedfast ice and 48% as having floating ice regimes (Grunblatt and Atwood 2014). The maximum ice thickness associated with the Grunblatt and Atwood (2014) SAR-based assessment of relative lake depth was 1.6 m. Thus, the technique provides an approximation of lakes shallower than 1.6 m and lakes deeper than 1.6 m. In terms of area, 55% of the ~900 km^2^ of lake surface water area in the watershed retained liquid water of unknown depth below the ice pan during the winter of 2009. Based on the IfSAR images and measured maximum lake depth points, a relative depth attribute (Fig. 3c) was created with 52% of the lakes in the FCW being shallow (<1.6 m deep), 32% being intermediate (>1.6 m and <~4.0 m), and 16% being relatively deep (>4.0 m).

### Lake connectivity

We differentiated four hydrologic connectivity classes based on field data collection and calculation of several minimum contributing area thresholds (Table 2; Figure S1, Fig.  3b). Isolated lakes were the most abundant (48.1%) of the four connectivity classes but were relatively small (<4 ha on average) and thus only represented 8.8% of total lake surface area. Temporarily connected lakes accounted for the greatest surface area (48.7%) and the second greatest in terms of number (38.1%). Lakes categorized as having a perennial connection or being a flow-through lake each accounted for less than 10% of the lake number but 19.3% and 23.3% of the area. These differences are also reflected in average lake sizes as the level of connectivity ranges from not connected to the most well connected going from isolated (3.7 ha), to temporary connection (26.6 ha), to perennial connection (47.5 ha), to flow-through (87.8 ha) in size.

### Lakes with islands and emergent vegetation

Lakes with islands and emergent vegetation potentially provide useful habitat to fish and wildlife species as they can be used to avoid predators and provide optimum foraging habitat. We manually identified 1197 lakes (27.4%) with islands and 332 lakes (7.6%) with emergent vegetation. Lakes with islands were distributed across all terrain units but were most prevalent in isolated or temporarily connected lakes (90%). Lakes with emergent vegetation were also well distributed in the watershed but preferentially occurred (52%) in lakes with a temporary connection.

### Lake dynamics

The majority of lakes (3651, 83.7%) remained stable (or below the detection limit of change) between 1985 and 2014 (Fig. 3d). However, these lakes only accounted for 37% of the surface area and had a mean lake area of 9.3 ha. The majority of the lakes mapped as stable (53%) were in the isolated connectivity class and 40% were classified as remnant drained lake basin marginal ponds. The remaining lakes predominantly increased their lake surface area with varying intensity (623, 14.3%), accounting for 55% of the total surface area in the watershed. Expanding lakes were predominantly classified as primary or secondary thermokarst or depression lakes (67%) and occurred most often in lakes classified as being isolated (22%) or having a temporary connection to a stream (46%). Dynamic lakes, or those mapped as exhibiting expansion and contraction, only accounted for 0.5% (22) of the population. The vast majority of the dynamic lakes occurred in primary or secondary thermokarst or depression lakes (95%) and in lakes with a temporary connection to a stream (86%). These lake types had the largest mean area (192 ha) and the lowest gradient (0.35 m) within a 100-m buffer surrounding the lake perimeter. Shrinking lakes account for only 1.5% (66) of the total number and 2.1% of the total area, with 48% of them being mapped as isolated.

### Applications for guiding research and informing management decisions

**Fig. 4:**
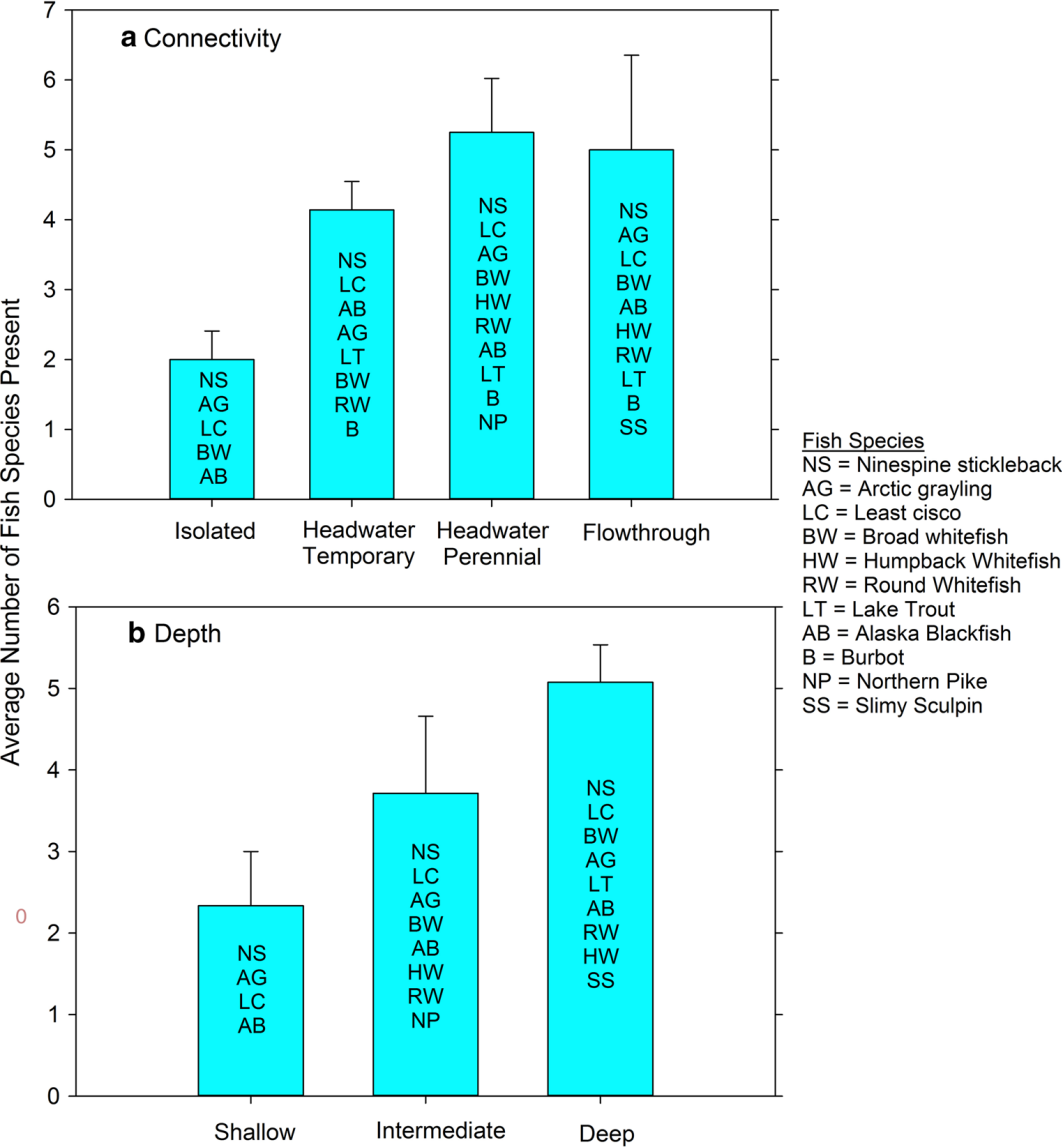
A comparison of the mean number of fish species present from a subset of lakes in the Fish Creek Watershed (*n* = 23, surveyed in the summers of 2014 and 2015) according to connectivity (**a**) and depth (**b**) classes (*error bars* are standard errors and individual fish species abbreviations are listed on each bar *top*-to-*bottom* from most common to least common)

**Fig. 5:**
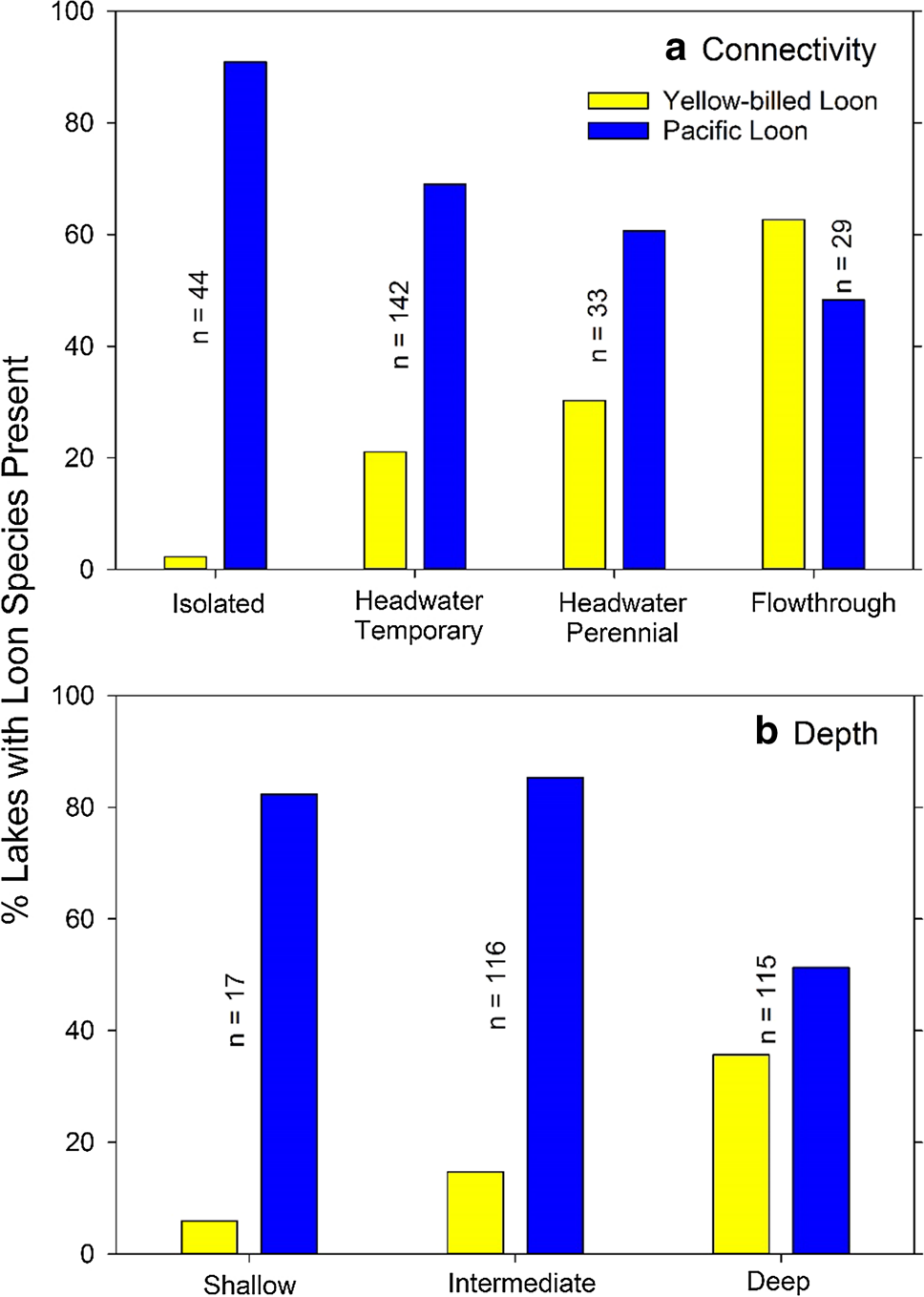
A comparison of yellow-billed and pacific loon presence from a subset of lakes in the Fish Creek Watershed (*n* = 248, lakes surveyed in early summer 2015) according to connectivity (**a**) and depth (**b**) classes

Lake-specific survey data of both fish and loon species presence was compared to the variables lake connectivity (four classes) and depth (three classes) in the geospatial database. For fish, a total of 11 species were captured in 23 lakes surveyed in 2014 and 2015. The number of species present increased from an average of 2 in isolated lakes up to an average of 5 in perennial headwater and flow-through lakes (Fig. 4a). Similar increases in fish species present were seen by depth class with average numbers of 2 in shallow, 4 in intermediate, and 5 in deep lakes (Fig. 4b). In the set of surveyed lakes, ninespine stickleback (*Pungitius pungitius*), an important prey species, were found in all lakes, while lake trout (*Salvelinus namaycush*), a top predator in the region, were found only in deep, connected lakes. Broad whitefish (*Coregonus nasus*), a species of particular subsistence value, were found in intermediate to deep lakes ranging across all connectivity classes (Fig. 4b). Over a much wider set of lakes (*n* = 248), Pacific loons (*Gavia pacifica*) were observed to be present in a majority of lake types with percentages declining with both increasing connectivity and depth classes (Fig. 5). An opposing pattern emerged from the comparison of lake classes for yellow-billed loon (*Gavia adamsii*) presence with this rarer species observed in only 2% of isolated and 6% of shallow lakes. Flow-through lakes, however, appeared to provide more optimal habitat, 63% of which had observed yellow-billed loons (Fig. 5a). Deep lakes also had a high portion of occupancy, 36%, of yellow-billed loons compared to other depth classes (Fig. 5b). Although these comparisons of lake classes to biological datasets are preliminary, the potential value of using the connectivity and depth classes and other information to guide management decisions for lake-rich Arctic landscapes is apparent.

**Fig. 6:**
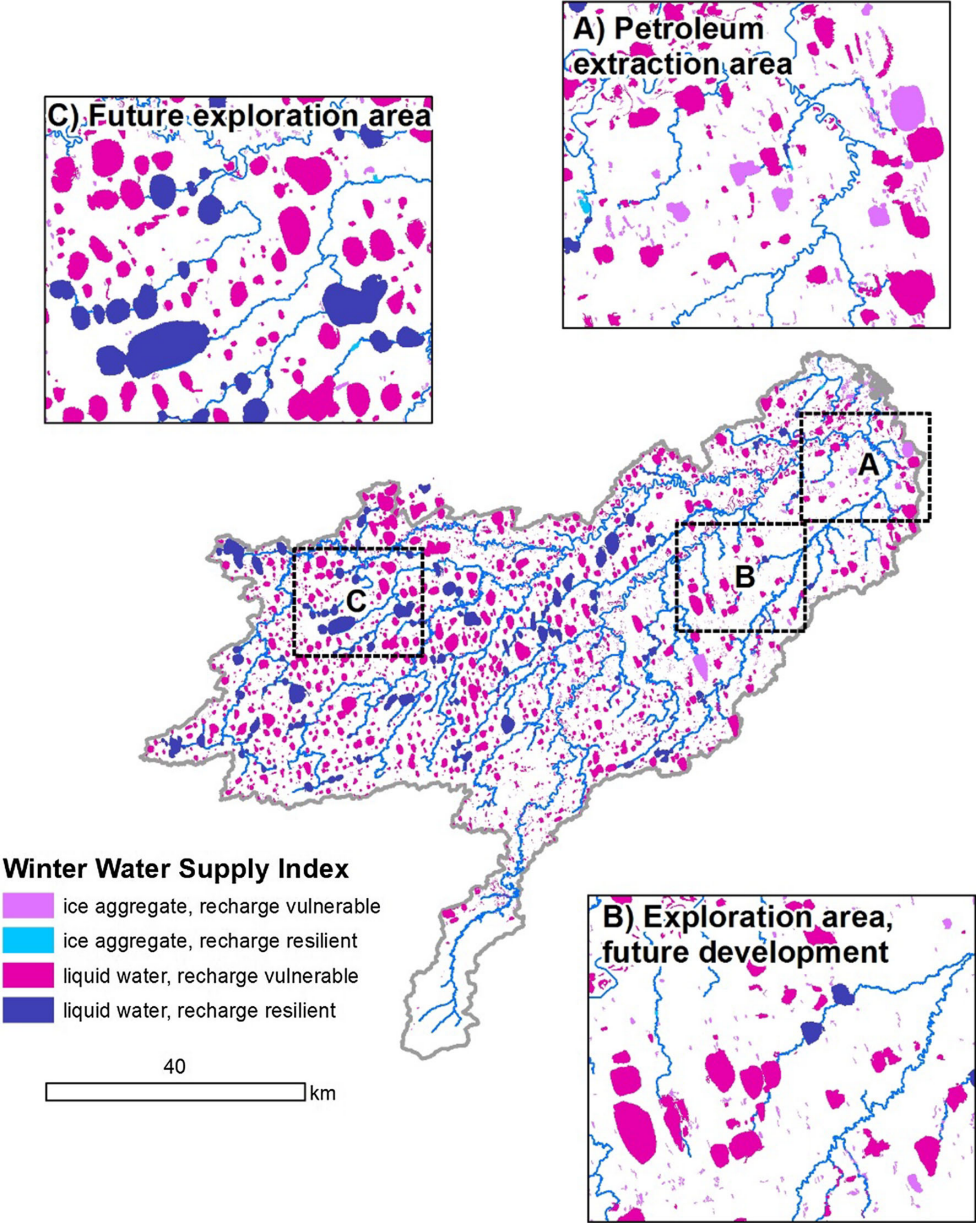
An example of the potential application of the lake-centric geospatial database for management decisions in the Fish Creek Watershed. Lake depth and connectivity classes were simplified and combined to make a Winter water supply index representing a combination of availability (ice chip source vs. liquid water source) and vulnerability to impact on downstream flows (based on connectivity)

Another potential use of the lake-centric geospatial database also involves the attributes of lake depth and connectivity but geared toward evaluating potential winter water use (Fig. 6). Here a four-class, Winter Water Supply Index was developed that could be used to assess resource availability [i.e., where only ice aggregate can be utilized (shallow lake class) and where liquid water and ice aggregate can be extracted (intermediate and deep classes)] as well as resource vulnerability [i.e., where lakes have low recharge potential (isolated and headwater temporary classes) and high recharge potential (headwater perennial and flow-through classes)]. Viewing lakes in the entire FCW using this index suggests numerous lakes with ice-aggregate availability in the lower watershed where development is currently occurring (Fig. 6a) and higher proportion of lakes with liquid water availability in the middle and upper watershed where future development is being planned (Fig. 6b, c). Localized variation in both resource availability and vulnerability is apparent throughout FCW, emphasizing the importance of considering physical lake attributes in addition to fish species presence for a more comprehensive and site-specific evaluation during water-use permitting.

## Discussion

Quantifying the number and distribution of lakes is important for Arctic lowland regions since lakes are a ubiquitous land cover type (Grosse et al. 2013) that serve an important role in regional habitat provision (Reist et al. 2006; Haynes et al. 2014a) and are an anthropogenic water source (Martin et al. 2007; Sibley et al. 2008). Comparing our lake-centric geospatial database with the Global Lakes and Wetland Database (GLWD: Lehner and Döll 2004) and one recent statewide product, the Alaskan Lake Database Mapped from Landsat Images (Sheng 2012) demonstrates the importance of our newly created lake dataset. The reported minimum mapping unit in both of these lake databases is 10 ha, whereas our minimum mapping unit is 1 ha. Thus, selecting lakes greater than 10 ha from our database shows that the GLWD underestimates the number of lakes greater than 10 ha by 70% and lake area by 33% in the FCW. The representation of lakes in the Sheng (2012) statewide database is an improvement; however, it underestimates the number of lakes greater than 10 ha by 3.0% and lake area by 1.4%. In addition, both databases do not capture lakes with a surface area smaller than 10 ha. Comparing the total lake number (4362) and surface area (900 km^2^) in our lake database with the GLWD (Lehner and Döll 2004) shows that 92% of the number and 40% of the lake area are missing and for the Sheng database (Sheng 2012) that 76% of the number and 19% of the lake area are not included when considering 1 ha as a minimum mapping unit. This has implications for the global distribution of lakes as the GLWD indicates that roughly 25% of the global lake population occurs in the northern high-latitude region (Lehner and Döll 2004). However, if lakes are commonly underrepresented in the Arctic in the GLWD, the relative role of Arctic lakes on mediating global process warrants further study and more accurate mapping (Palton et al. 2015). Thus, the IfSAR-derived lake data layer provides an improvement over previously available datasets for the study area since it is more comprehensive and better suited for research and management needs in the watershed.

Going beyond typical classification systems that tend to map lakes as a single, uniform class is important for better characterizing these ubiquitous Arctic land cover types. Our watershed-based, lake-centric geospatial database does this by providing information beyond simple lake distribution and lake surface area attributes. The geospatial dataset includes information on shape complexity, elevation, bluff characteristics, lake type, hydrologic connectivity, relative depth, prey avoidance features (islands), emergent vegetation, landscape position, surface area dynamics, and the surrounding terrestrial ecotypes. In all, the dataset contains 22 descriptive attributes for each lake. We provide an accuracy assessment of the various attributes in the lake-centric geospatial database in Table 1. Rigorously and unambiguously determining the overall accuracy of the interaction of various attributes in a GIS framework is challenging when combining data from multiple data sources (Foody and Atkinson 2003). However, efforts such as this are still extremely useful for better understanding and categorizing natural and human-modified systems. Similar geospatial databases have been developed for lakes and wetlands located in tropical and temperate regions that have been used to manage aquatic resources (Rebelo et al. 2009; Soranno et al. 2010; Panigrahy et al. 2012; Olmanson et al. 2013). However, our lake-centric geospatial database is the first such comprehensive example developed for lakes located in the Arctic and can be used as a guide for developing similar products across other lake-rich Arctic regions.

The lake-centric geospatial database may also be used to potentially explain observations of changing Arctic lakes. Several remote sensing-based studies have been conducted in the Arctic that track changes in lake surface area over decadal time scales (i.e., Smith et al. 2005; Riordan et al. 2006; Jones et al. 2011; Lantz and Turner 2015). The driving forces guiding changes in lake surface area extent are often difficult to interpret since lake-specific information is typically lacking. In the FCW, lakes were primarily stable during the 1985 to 2014 observation period. However, the most dynamic lakes were mapped as primary or secondary thermokarst or depression lakes with actively expanding margins into permafrost terrain and with a temporary hydrologic connection, whereas nearly half of the shrinking lakes in the FCW were mapped as being hydrologically isolated. In terms of hydrologic connectivity, our assessment indicates that about 50% of the lakes in the FCW are isolated but that 91% of the surface area is hydrologically connected during some portion of the summer season. In terms of a perennial streamflow connection, our assessment builds upon previous research conducted on a subset of lakes in the FCW that indicated that 66% of the surface area in the watershed was hydrologically connected the entire summer (Arp et al. 2012a), which is also similar to estimates of connectivity in lakes in the MacKenzie River Delta region (Lesack and Marsh 2007). Our analysis indicates that only 43% of the area and less than 10% of the number of lakes in the FCW have a perennial stream connection. While this assessment is limited in the number of lakes surveyed, it may provide an indication that lakes in the FCW may be more vulnerable to climate and land-use change pressures than previously thought.

At the regional scale, the value of this aquatic habitat template will help guide more informed, spatially explicit management and policy for this Arctic watershed in the NPR-A where both land-use and climate changes are observable now and are expected in the foreseeable future. Much of the biologically relevant landscape heterogeneity in freshwater habitats in the FCW are due to ice regimes determined by water depth relative to ice thickness (i.e., bedfast ice vs. floating ice) and watershed position that determines surface connectivity (Lesack and Marsh 2010; Arp et al. 2012a). Initial comparisons of lake connectivity and depth classes to fish species present as surveyed in 23 lakes show increasing fish species richness as hydrologic connectivity increases (Fig. 4a). This generally follows results from other Arctic Coastal Plain studies in which lakes with a permanent channel connection had the greatest fish species richness, while fish presence was more limited in lakes with temporally restricted access (Haynes et al. 2014b; Laske et al. 2016). A similar pattern was also observed with respect to lake depth and species richness in which shallow lakes that freeze solid with bedfast ice in the winter only support a few fish species during the summer (Fig. 4b). Making the same lakes comparison using presence data of two species of loons as surveyed in 248 lakes shows some of the differences in habitat selection by yellow-billed and pacific loons in association with forage resources (number of fish species present in lake), lake depth, and lake connectivity. These comparisons also suggest the potentially interspecific exclusion of pacific loons in lakes used by yellow-billed loons for breeding (Fig. 5). Results from other Arctic Coastal Plain studies, in which yellow-billed and pacific loon nesting habitats were studied (Earnst et al. 2006; Haynes et al. 2014a), agree with our preliminary findings of increasing lake depth and connectivity being correlated with yellow-billed loon presence, while Pacific loons showed the opposite trend (Fig. 5). Haynes et al. (2014a) found strong indications of interspecific competition between yellow-billed and pacific loons occupying lakes within the Arctic Coastal Plain and our current loon dataset will be tested to determine if loons in the FCW show the same degree of potential interspecific competition. The important advancement that the development of this lake-centric geospatial database for an entire Arctic watershed provides is that once these fish and loon datasets are rigorously analyzed and occupancy models developed, it will afford us the ability to make wider landscape determinations of populations and habitat distribution. The lake-centric geospatial database described also proved valuable during the planning phase of an ongoing lake trout (*Salvelinus namaycush*) study taking place in the FCW. The physical attributes, depth and connectivity, are thought to be the critical factors explaining the presence of particular fish species (Laske et al. 2016). These attributes were utilized during site selection and ultimately facilitated field sampling efforts. The lake-centric geospatial database is also being used to better understand the zooplankton communities (crustacean and rotifers) of lakes in the FCW. Preliminary analyses show that large-bodied crustaceans (*Daphnia*, calanoid copepods) are restricted to small, shallow lakes with low lake connectivity, while small-bodied crustaceans (*Bosmina*) and rotifers dominate large, deep lakes with high lake connectivity (Beaver personal communication). Thus, our lake-centric geospatial database for the FCW provides a template for better organizing this complex lake-rich watershed, designing monitoring programs and scientific studies, and ultimately guiding management decisions based on how aquatic habitats function at a relevant landscape scale.

The lake-centric geospatial database provides the necessary data to make more informed decisions regarding aquatic resource management in the NPR-A. The coupling of geospatial data with biological and physical lake information improves the ability to make informed land management decisions by facilitating a more comprehensive evaluation of resource value and risk when considering various land-use activities that could occur to support oil and gas exploration and development. For example, current guidelines regarding industrial water use are based solely on fish species found in a lake (USDOI BLM 2013). However, considering multiple lake attributes as well as fish species will contribute to a more robust site-specific evaluation rather than simply applying a broad regional formula to determine outcomes. Figure 6 provides an example of the potential application of the database for management decisions in the FCW. Our lake-centric geospatial database can also be used to better understand how watershed-scale hydrology might be impacted given ongoing and projected changes to the Arctic terrestrial hydrology system (Bring et al. 2016) and how this may in turn impact wintertime activities associated with oil and gas exploration and development. Such a classification scheme could be useful in guiding industry and resource managers as to what lakes can currently supply relative to potential impacts of extraction however remains to be validated in the field. However, we caution here that our results related to winter water use should be considered preliminary and that further investigation is necessary to determine the resilience and vulnerability of specific lakes as ice chip and/or water sources.

While the lake-centric geospatial database developed in this study is novel and will provide useful information going forward, it is primarily static in nature and thus inherently limiting. Freshwater Arctic ecosystems are dominated by lakes with dynamic regimes of connectivity, water balance (Bowling et al. 2003; Arp et al. 2012a), morphology (Jones et al. 2009), and ice cover duration and thickness (Arp et al. 2012b, 2015b) that vary spatially and temporally. Habitat classification is often seen as a static inventory by mapping landscape units without capturing the inherent dynamic nature that is increasingly important to consider in both science and management. Many attributes of our lake-centric geospatial database are expected to change over time. For example, lake connectivity to stream networks may expand and contract regionally in wetter or drier summers, respectively, and individual lakes may also respond asynchronously with changes in morphology (i.e., partial or complete catastrophic drainage) or in association with flooding from adjacent rivers. Similarly, we expect that lake depth classes will shift not only according to moisture conditions, but also, and perhaps more so, with changing ice thickness that may cause fewer lakes to develop bedfast ice regimes (Arp et al. 2012b). A lack of current understanding of the linkages among climate change, physical drivers, and biological responses at relevant scales presents managers and other stakeholders with great uncertainty (Streever et al. 2011). Such dynamics present a limitation with using systems of habitat classification, yet also an opportunity to build dynamics into attributes and classes and use these classes as a framework for monitoring and detecting habitat change over time. In these instances, the use of hydrological models is valuable for understanding temporally and spatially varying climate–permafrost–hydrology interactions at the watershed scale. Going forward, the lake-centric geospatial database will be used to guide process-based, spatially distributed hydrological model development in data-scarce remote Arctic watersheds. This database and its results can support model parameterization and evaluation, and inform sensitivity analyses, especially when models are applied across regional domains. Field measurements combined with an overall lake-centric geospatial database of the FCW watershed presents an effective proxy basin to assess how climate and land cover changes may affect similar Arctic watersheds. Specifically, understanding the temporal variability of hydrologic connectivity among rivers, streams, and lakes and how this affects fish migration and habitat use, will in turn aid regional stakeholders in their planning. The opportunities afforded by the development of the detailed lake-centric geospatial database for the FCW and its utility in regional research strategies, management decisions, and model development may assist in developing similar and appropriate datasets for other lake-rich Arctic landscapes.

## Conclusion

In this study, we describe a lake-centric geospatial database developed for an Arctic watershed that is currently experiencing climate and land-use change pressures. The Fish Creek Watershed (FCW) is located on the Arctic Coastal Plain of northern Alaska where numerous climate change effects have been observed. At the same time, it is located in the National Petroleum Reserve in Alaska (NPR-A), where active oil and gas exploration and development has occurred during the past decade, making it an important and interesting study watershed for human–landscape interactions in the Arctic. The geospatial database provides information on lake morphometry, hydrologic connectivity, surface area dynamics, surrounding terrestrial ecotypes, and other important habitat characteristics for more than 4000 lakes in the 4900 km^2^ FCW. The lake-specific information available in the geospatial dataset is useful for guiding research questions, enhancing model development, and informing management decisions in the FCW, the NPR-A, and eventually in lakes across the Arctic that have a global importance.

## Electronic supplementary material

Below is the link to the electronic supplementary material.

Supplementary material 1 (PDF 222 kb)
