# Supplementary material for: A lake-centric geospatial database to guide research and inform management decisions in an Arctic watershed in northern Alaska experiencing climate and land-use changes
Source: Ambio. 2017 Mar 25;46(7):769–86. doi: 10.1007/s13280-017-0915-9 (PMC5622880; doi:10.1007/s13280-017-0915-9)
Supplement: Supplementary file 1 — Supplementary material 1 (PDF 222 kb) [file 13280_2017_915_MOESM1_ESM.pdf]

Ambio

Electronic Supplementary Material

This supplementary material has not been peer reviewed.

Title: A lake-cover classification to guide research and inform management decisions in an arctic watershed in northern Alaska experiencing climate and land-use changes

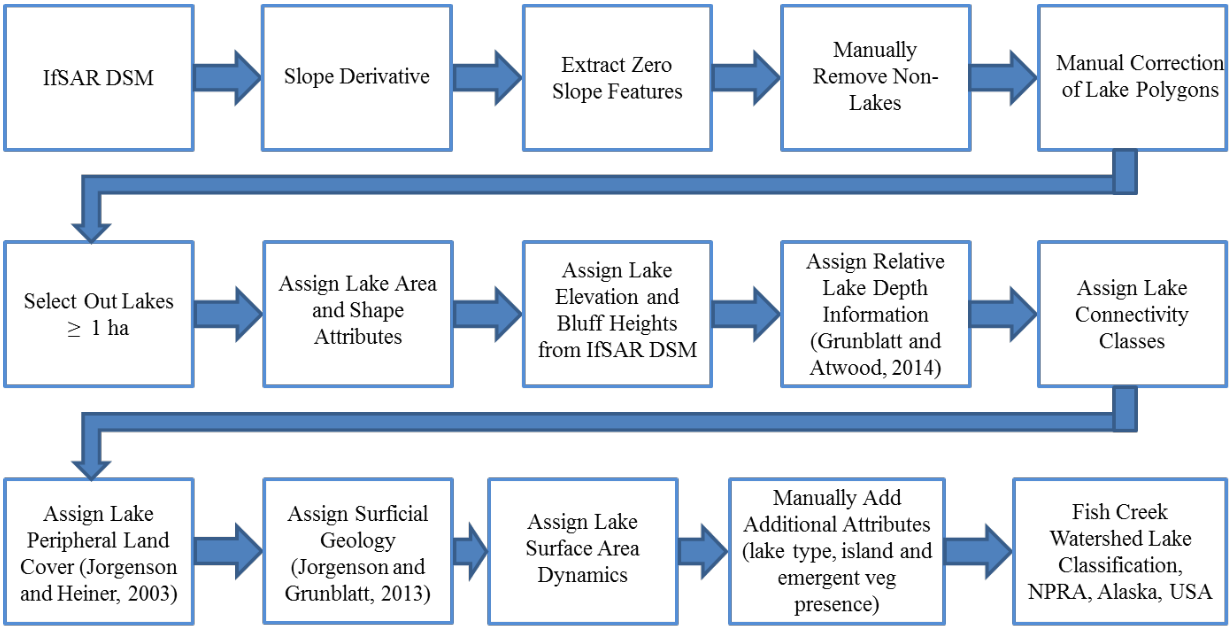

Figure S1. Workflow diagram describing the steps involved in creating the lake cover classification for the Fish Creek Watershed, NPRA, Alaska, USA.

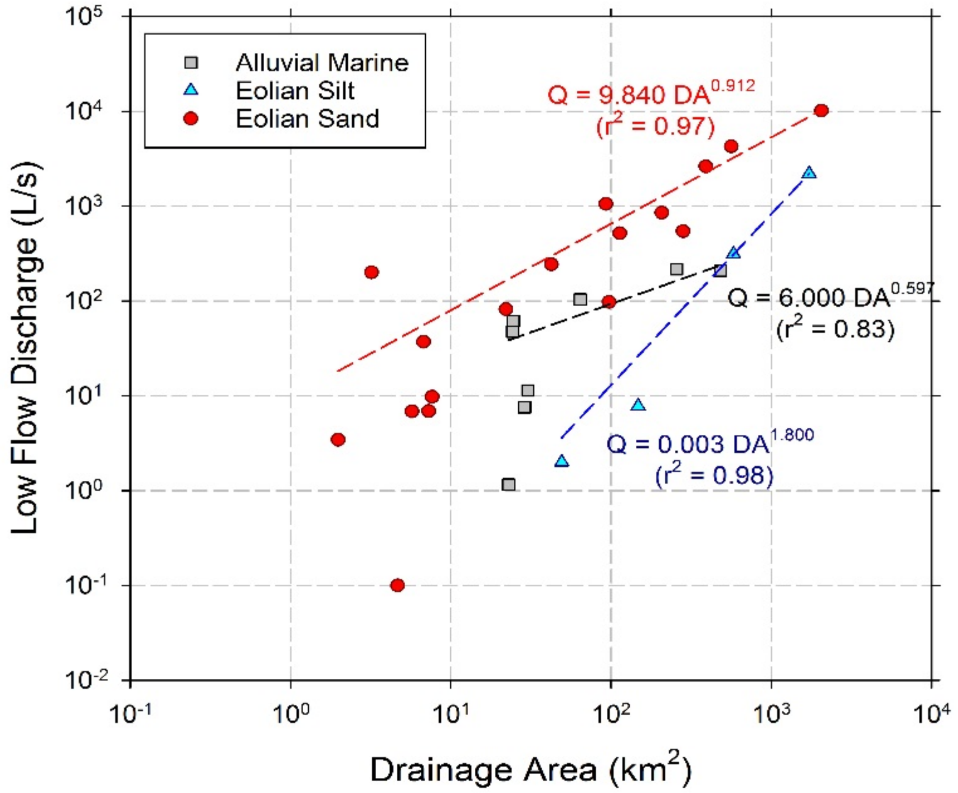

Figure S2. Relations between discharge and drainage area measured in mid-July 2015 during a prolonged dry period when stream-lake connectivity was most limited. Measurements are partitioned by surface geology to develop lake connectivity criteria specific to each region in the Fish Creek Watershed (Table 2).

Appendix S1. Shapefile attribute dictionary for the FCW_lake_classification_2016.shp

DOI: http://dx.doi.org/10.5066/F7H70CXB

FID: Arbitrary ID for each polygon

Shape: Vector feature description. Lake polygons were derived from a 5m resolution IfSAR digital surface model by calculating a zero slope. Each feature was expanded by one pixel around the entire perimeter since all waterbodies were truncated by this during the slope calculation. Lakes >=1ha were manually extracted from the dataset and their perimeters further corrected using 2002 CIR orthophotography.

ID: Permanent ID for each polygon.

Longitude: Lake centroid in WGS84

Latitude: Lake centroid in WGS84

Perim_km: Lake perimeter in kilometers

Area_sq_km: Lake surface area in square kilometers

Hectares: Lake surface area in hectares

Shape_ind: Shape index or shoreline complexity measure for polygon features. This is equal to the perimeter divided by four times the square root of its area. The more fractal an object is the higher its shape index or complexity.

Elev_masl: Lake surface elevation in meters as derived from IfSAR digital surface model

Surf_geo: Surface geology classification for each lake derived from Jorgenson Landscape Ecological Mapping classification

- 1: Delta
- 2: Floodplain
- 3: Eolian Sand
- 4: Eolian Silt
- 5: Marine Sand

Lake_type: Manually interpreted lake type. This includes relatively broad classes that are aimed at describing lake origin and history.

- 1: Remnant drained lake basin pond
- 2: Primary or secondary thermokarst or depression lake
- 3: Oxbow lake
- 4: Deltaic lake
- 5: Wellhead pond

- 6: Collapsed pingo pond

Depth_cat: This refers to whether a lake is a grounded or floating ice lake according to the 2009 SAR classification based on Grunblatt and Atwood SAR classification. Lakes with less than 1% floating ice conditions in 2009 were classified as grounded ice.

- 0: Grounded Ice Lake
- 1: Floating Ice Lake

Unfrz_perc: This refers to the percent of the lake area that was floating ice as derived from the Grunblatt and Atwood 2009 SAR classification except where noted below.

Rel_depth: Indicates whether a lake feature is shallow (grounded ice), intermediate (floating ice with no deep central pool), or deep (floating ice with deep central pool). The distinction between the intermediate and deep classifications was manually interpreted from the 2002 CIR orthophotography and aided by a compilation of ~200 maximum lake depths in the watershed, in addition to visual interpretation of depths greater than ~4 m in the 2002 CIR orthophotography.

Connectiv: Indicates how hydrologically connected each lake feature is. Based on surficial geology and specific hydrology relative to minimum contributing areas and flow data collected during July 2015. Minimum contributing areas relative to these four classes, among the five surficial geology types, were derived using the ArcGIS Hydrology toolset.

- 1: Isolated
- 2: Ephemeral Connection (Typically only connected early in season or during wet year)
- 3: Perennial Connection (Primarily outflow)
- 4: Flow through (Perennial inflow and outflow)

Mean_b_hgt: The mean height of a lake bluff based on a 5 m buffer around each lake.

Min_b_hgt: The minimum height of a lake bluff based on a 5 m buffer around each lake.

Max_b_hgt: The maximum height of a lake bluff based on a 5 m buffer around each lake.

100m_gradi: The difference between the surface elevation of a lake and the minimum elevation in a 100 m buffer around the lake perimeter.

Dist_cst: Distance to coast in kilometers as determined from a polyline adjacent to the FCW delta. This is measured as a straight line distance.

Islands: Manual interpratation of the lakes with islands based on the 2002 CIR orthophotography.

- 0: No
- 1: Yes

Emerg_veg: Manual interpretation of lakes with visible emergent vegetation (most likely *Arctophila fulva*) based on the 2002 CIR orthophotography.

- 0: No
- 1: Yes

Dom_Ecosys: The dominant ecosystem type in a 90 m buffer around each lake perimeter derived from the TNC landcover classification. These cover types include: coastal barren, coastal grass and dwarf shrub tundra, coastal water, coastal wet sedge tundra, lowland lake, lowland moist sedge shrub tundra, lowland wet sedge tundra, riverine barrens, riverine moist sedge shrub tundra, riverine waters, riverine wet sedge tundra, upland dryas dwarf shrub tundra, upland low shrub birch willow tundra, upland moist sedge shrub tundra, upland shrubby tussock tundra, and upland tussock tundra.

LSAD: Lake surface area dynamics were extracted from a dense time series of Landsat TM, ETM+, and OLI imagery available from 1985 to 2014. All cloud-free pixels were analyzed over this 30 year period and classified as either water or non-water. This information was aggregated for the entire time series and trends in lake surface area were categorized over the entire time series as (1) expanding lakes, (2) shrinking lakes, (3) dynamic lakes, and (4) stable lakes.
